# Clinical implication of a quantitative frailty assessment tool for prognosis in patients with urological cancers

**DOI:** 10.18632/oncotarget.24712

**Published:** 2018-04-03

**Authors:** Osamu Soma, Shingo Hatakeyama, Teppei Okamoto, Naoki Fujita, Teppei Matsumoto, Yuki Tobisawa, Tohru Yoneyama, Hayato Yamamoto, Takahiro Yoneyama, Yasuhiro Hashimoto, Takuya Koie, Shigeyuki Nakaji, Chikara Ohyama

**Affiliations:** ^1^ Department of Urology, Hirosaki University Graduate School of Medicine, Hirosaki, Japan; ^2^ Department of Advanced Transplant and Regenerative Medicine, Hirosaki University Graduate School of Medicine, Hirosaki, Japan; ^3^ Department of Social Medicine, Hirosaki University School of Medicine, Hirosaki, Japan

**Keywords:** frailty, gait speed, urological cancer, prostate cancer, urothelial carcinoma

## Abstract

**Objectives:**

Optimal tools for evaluating frailty among urological cancer patients remain unclear. We aimed to develop a quantitative frailty assessment tool comparing healthy individuals and urological cancer patients, and investigate the clinical implication of quantitative frailty on prognosis in urological cancer patients.

**Results:**

Gait speed, hemoglobin, serum albumin, exhaustion, and depression were significantly worse in patients with all types of cancers than in pair-matched controls. Frailty discriminant score (FDS) showed clear separation between controls and urological cancer patients, and significant association with the Fried criteria. Overall survivals were significantly shorter in patients with a higher score (>2.30) than in those with a lower score among nonprostate cancer (bladder, upper tract urothelial carcinoma, and renal cell carcinoma) patients. In prostate cancer patients, overall survivals were significantly shorter in patients with a higher score (>3.30) than in those with a lower score.

**Conclusions:**

FDS was significantly associated with frailty and prognosis in urological cancer patients. This tool for frailty assessment can help patients and physicians make more informed decisions. Further validation study is needed.

**Materials and Methods:**

Total 605 urological cancer patients presenting to our hospital underwent a prospective frailty assessment. Controls were selected from 2280 community-dwelling subjects. Frailty was assessed via physical status, blood biochemical tests, and mental status. We compared frailty variables between pair-matched controls and urological cancer patients. We developed FDS using frailty variables, and compared with the Fried criteria. The influence of FDS on overall survivals was investigated by Kaplan-Meier analysis and Cox regression analysis.

## INTRODUCTION

The concept of frailty has become recognized as a key factor in cancer treatment [1, 2]. Physical parameters, mental status, comorbidities, and serum biochemical parameters are indicators of functional capacity and frailty syndrome [3–6]. Recently, there has been growing interest in measuring patients’ frailty using several tools to understand the functional and physiologic heterogeneity among the elderly with urological diseases [6–8]. However, no consensus exists regarding which items or tools should be used to measure frailty. Currently, there are two main models to assess frailty, including the frailty phenotype (Fried criteria) [9] and frailty index (Rockwood index) [10]. In the Fried criteria, the criteria scored weight loss, grip strength, self-reported exhaustion, walking speed, and activity level. The Rockwood index counts impairments, including symptoms, signs, diseases, and disabilities. Both models have been widely used for research in the perioperative setting, including modified methods [1, 6–8, 11, 12]. However, a full geriatric assessment in all candidates is time-consuming and not feasible for clinical practice. Therefore, an easy and simple tool to evaluate frailty is required.

We hypothesized that differences in key parameters between healthy individuals and cancer patients might be optimal for quantitative measurement of cancer-related frailty. We compared physical capabilities (handgrip weakness and slowed walking speed), blood biochemical tests (serum albumin, renal function, and hemoglobin), and self-reported exhaustion and depression between community-dwelling healthy individuals and cancer patients. We aimed to develop a quantitative assessment tool for frailty and to investigate the clinical implication of the quantitative frailty score on prognosis in urological cancer patients.

## RESULTS

### Background comparison between pair-matched controls and patients

**Figure 1:**
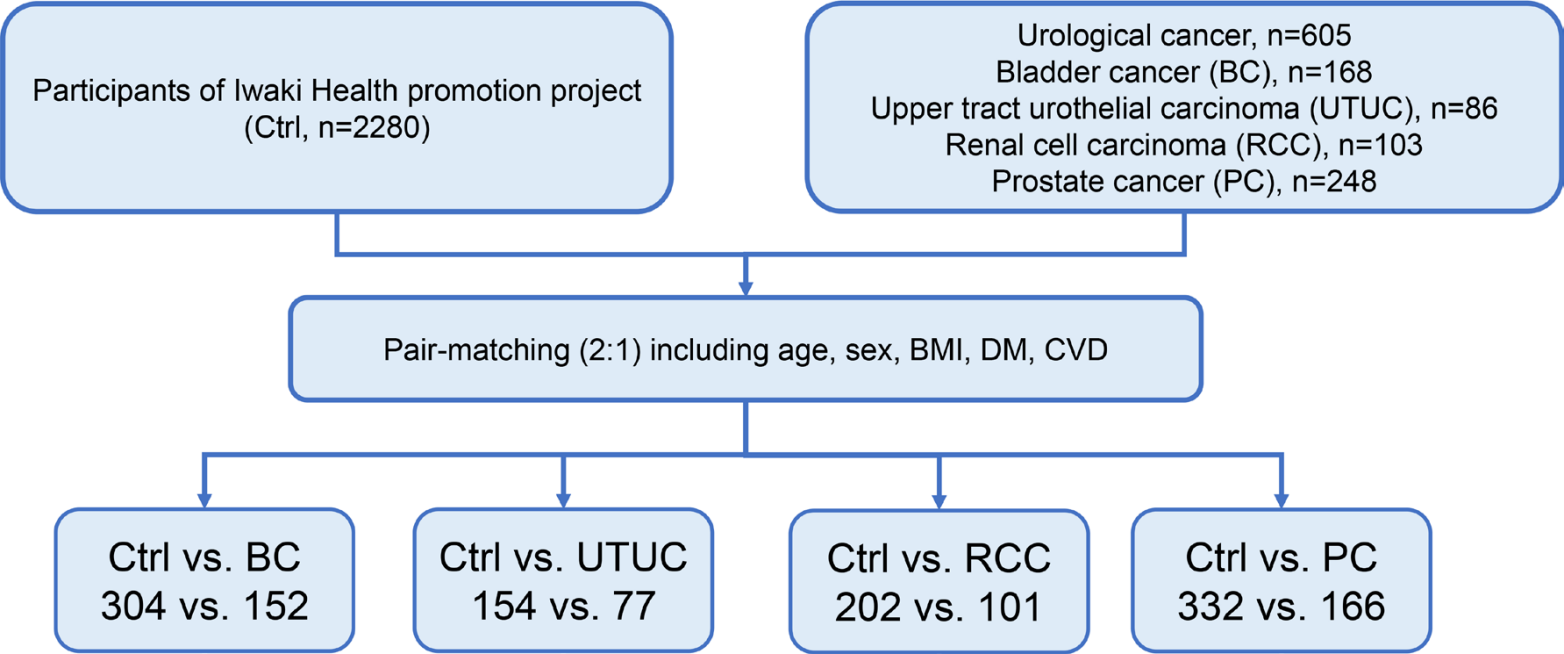
Patient selection for pair-matching We used propensity score matching to select two pair-matched controls and one urological cancer patient. Propensity scores were calculated using logistic analysis, and they accounted for age, sex, BMI, and presence of comorbidities (DM and/or CVD).

**Figure 2:**
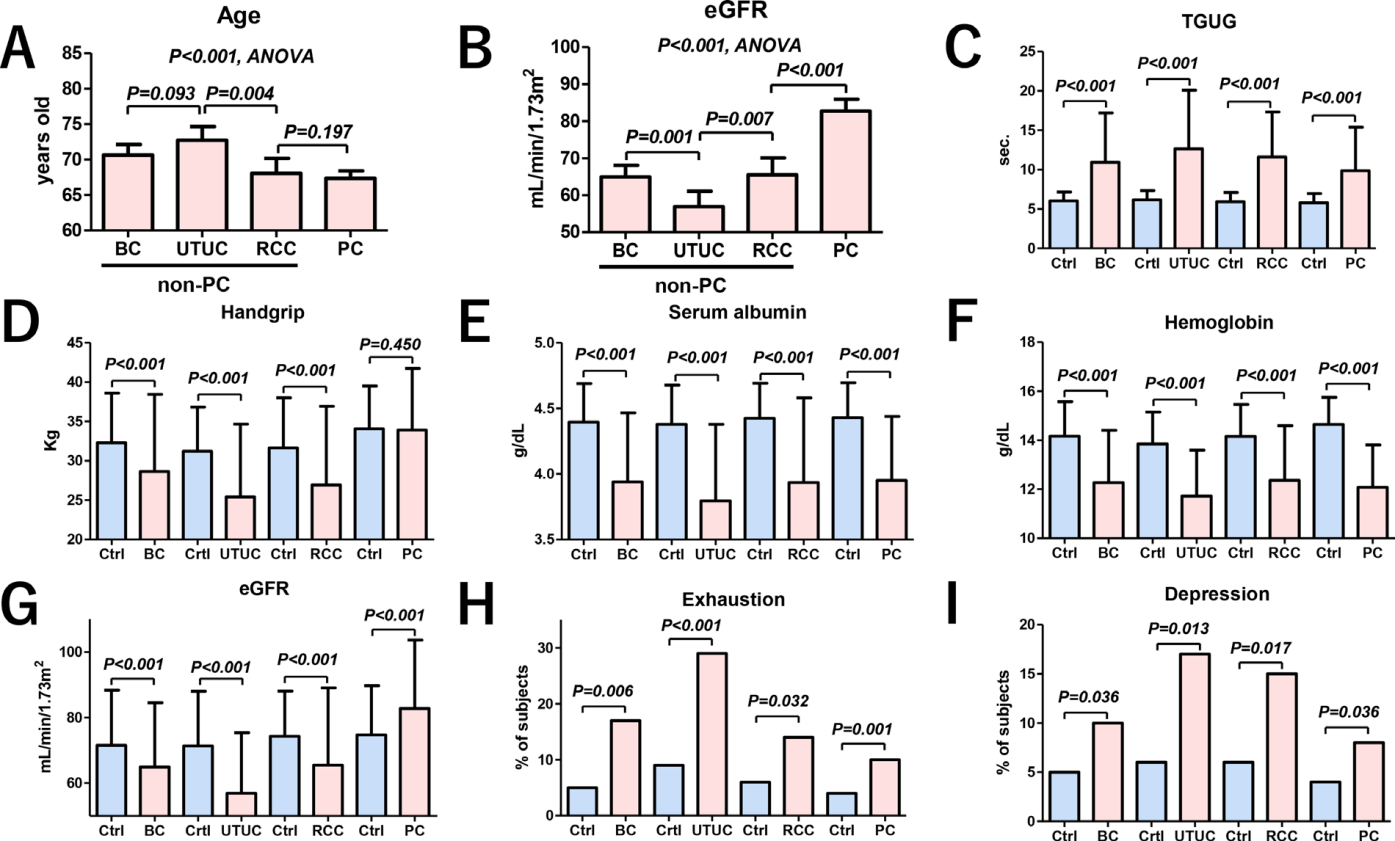
Variables comparison between the pair-matched controls and urological cancer patients Age (**A**) and eGFR (**B**) were significantly different among the urological cancer patients (*P* < 0.001, ANOVA). Patients with PC had a significantly higher eGFR than patients with BC, UTUC and RCC (B, *P* < 0.001, ANOVA). TGUG was significantly slower in patients with all types of urological cancers than in controls (**C**). Handgrip strength was significantly weaker in patients with BC, UTUC, and RCC than in controls, whereas no difference was observed in patients with PC (**D**). Serum albumin (**E**) and hemoglobin (**F**) were significantly lower in patients with all types of urological cancers than in controls. Renal function was significantly lower in patients with BC, UTUC, and RCC than in controls, whereas it was significantly higher in those with PC (**G**). The numbers of patients with exhaustion (**H**) and depression (**I**) were significantly higher in those with all types of urological cancers than in controls. Ctrl: controls.

Backgrounds of all subjects in our study are summarized in the Table 1. Bladder cancer (BC), upper tract urothelial carcinoma (UTUC), renal cell carcinoma (RCC), and prostate cancer (PC) occurred in 168, 86, 103, and 248 patients, respectively. To adjust backgrounds, we used propensity score matching in the cancer patients and pair-matched controls (Figure 1). After the matching, there were no significant differences in age, sex, body mass index (BMI), history of cardiovascular disease (CVD), diabetes mellitus (DM) between the two groups (Table 2). Age (Figure 2A) and estimated glomerular filtration rates (eGFR) (Figure 2B) were significantly different among the urological cancer patients (*P* < 0.001). The timed get-up and go test (TGUG) (Figure 2C), serum albumin (Figure 2E), hemoglobin (Figure 2F), presence of exhaustion (Figure 2H), and depression (Figure 2I) were significantly worse in patients with all types of cancers than in controls. Handgrip strength (Figure 2D) and renal function (Figure 2G) were significantly lower in non-PC (BC, UTUC, and RCC) patients than in controls, whereas renal function was significantly higher in patients with PC than in controls. Handgrip strength was not significantly different between the controls and patients with PC.

**Table 1:** Background of subjects

	Ctrl	Urological Cancers
*n*	2280	605
Age, years	55 ± 15	70 ± 8.7
Sex (male), *n*	874 (38%)	495 (85%)
ECOG-PS (>1)		34 (5.6%)
Body mass index (BMI, kg/m^2^)	23 ± 3.4	24 ± 8.2
Diabetes mellitus (DM), *n*	182 (8.0%)	95 (16%)
Cardiovascular disease (CVD), *n*	196 (8.6%)	74 (13%)
Handgrip strength (Kg)	28 ± 8.4	30 ± 9.4
TGUG (sec.)	5.5 ± 1.2	11 ± 5.5
Exhaustion (yes), *n*	139 (6%)	89 (15%)
Depression (yes), *n*	150 (7%)	75 (12%)
Serum Albumin (g/dL)	4.5 ± 0.3	3.9 ± 0.5
eGFR (mL/min/1.73m^2^)	80 ± 16	71 ± 22
Hemoglobin (g/dL)	14 ± 1.5	12 ± 1.9
Type of urological cancers, *n*		
Bladder cancer (BC)		168 (28%)
Upper tract urothelial carcinoma (UTUC)		86 (14%)
Renal cell carcinoma (RCC)		103 (17%)
Prostate cancer (PC)		248 (41%)
Metastatic disease, *n*		95 (16%)

TGUG: timed get-up and go test. TGUG is a simple test used to assess a person’s mobility and requires both static and dynamic balance. It uses the time that a person takes to rise from a chair, walk three meters, turn around, walk back to the chair, and sit down. CVD was defined as a positive history of cardiac surgery, angina, myocardial infarction, stroke, or taking any cardiotonic agents and/or coronary vasodilators.

**Table 2:** Background of pair-matched subjects

	Ctrl	BC	*P* value	Ctrl	UTUC	*P* value	Ctrl	RCC	*P* value	Ctrl	PC	*P* value
*n*	304	152		154	77		202	101		332	166	
Age (years)	71.0	71.0	*0.976*	73.0	73.0	*0.761*	69.0	69.0	*0.491*	66.0	67.0	*0.882*
Sex (Male)	78%	78%	*1.000*	69%	68%	*0.843*	69%	73%	*0.472*	100%	100%	*1.000*
BMI (kg/m^2^)	23.2	22.8	*0.698*	23.2	22.8	*0.698*	23.2	22.8	*0.698*	23.2	22.8	*0.698*
Diabetes mellitus	17%	18%	*0.862*	21%	22%	*0.911*	20%	19%	*0.837*	17%	14%	0.330
CVD	20%	21%	*0.871*	17%	17%	*0.967*	15%	11%	*0.269*	17%	14%	*0.423*
T1		30%			3.9%			50%			95%	
T2-3		64%			81%			42%				
T4		7%			16%			7.9%			5.4%	
Metastatic		9%			31%			29%			16%	

### Development of quantitative frailty score

**Figure 3:**
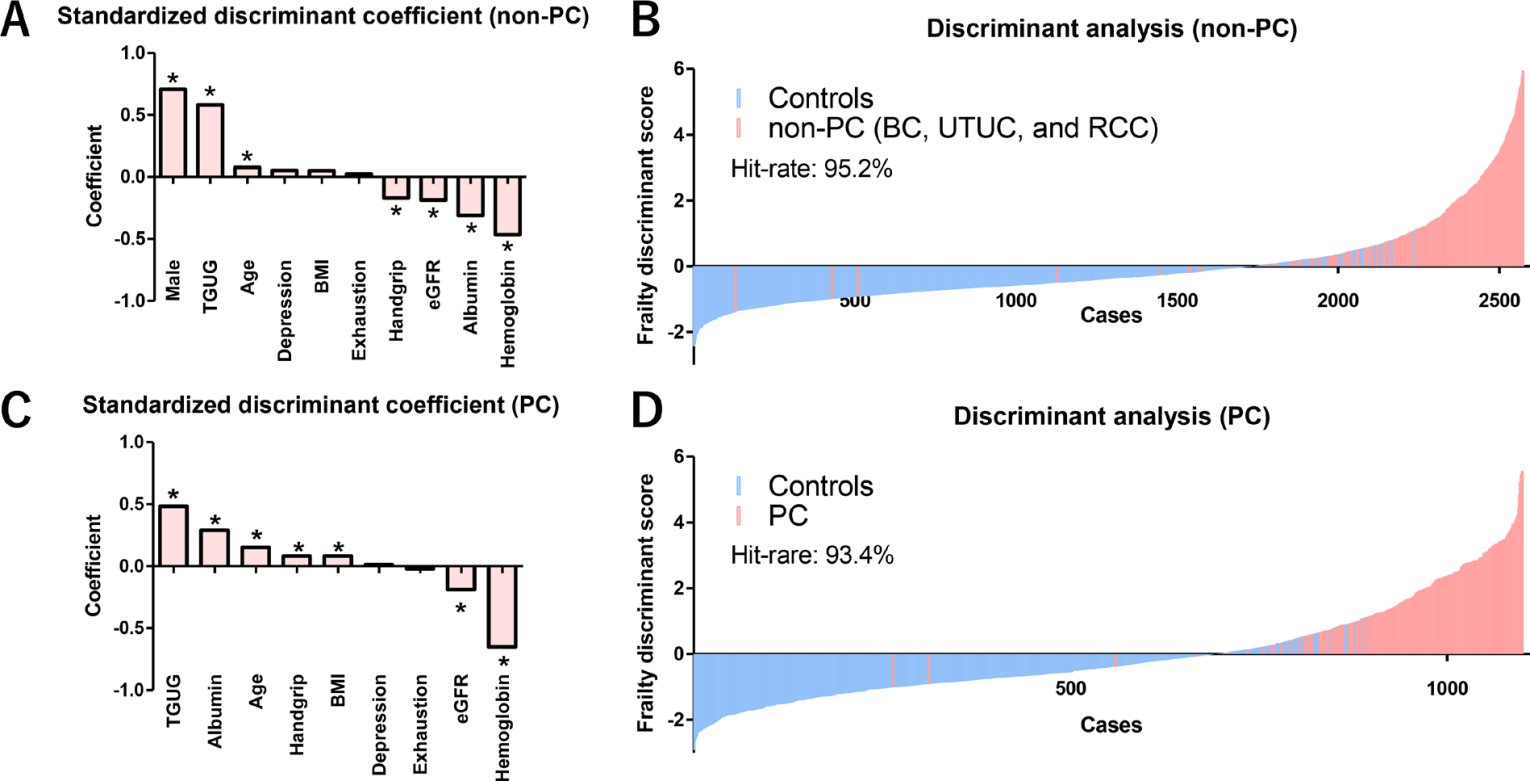
Impact of assessment items on frailty and waterfall plot of FDS for non-PC and PC patients Controls for discriminant analysis for non-PC patients included all 2280 control subjects. Standardized discriminant coefficient values showed that male sex (*P* < 0.001), TGUG (*P* < 0.001), and age (*P* = 0.012) were associated positively with frailty, whereas handgrip strength (*P* < 0.001), eGFR (*P* < 0.001), serum albumin (*P* < 0.001), and hemoglobin (*P* < 0.001) were associated negatively with frailty in the non-PC (BC, UTUC, and RCC) patients (**A**). Waterfall plot of the FDS showed a clear separation between the controls (*n* = 2280) and the non-PC patients. The hit-rate of FDS for the non-PC patients was 95.2% (**B**). Controls for discriminant analysis for PC patients included 874 male subjects in controls. TGUG (*P* < 0.001), serum albumin (*P* < 0.001), age (*P* < 0.001), handgrip strength (*P* = 0.040), and BMI (*P* = 0.041) were associated positively with frailty in the PC patients, whereas eGFR (*P* < 0.001) and hemoglobin (*P* < 0.001) were associated negatively with frailty in the PC patients (**C**). Waterfall plot of the FDS showed a clear separation between the male controls (*n* = 874) and PC patients. The hit-rate of FDS for the PC patients was 93.4% (**D**). ^*^Statistically significant (*P* < 0.05).

We developed two frailty discriminant formulas for PC and non-PC patients. Frailty discriminant formulas for non-PC and PC patients were obtained as follows: non-PC = (6.8698 + age × 0.0053 + sex × 1.4794 + BMI × 0.0105 + handgrip × −0.0209 + TGUG × 0.1993 + exhaustion × 0.0876 + depression × 0.2005 + albumin × −0.9037 + eGFR × −0.0112 + hemoglobin × −0.2868), and PC = (5.6418 + age × 0.0110 + BMI × 0.0267 + handgrip × 0.0094 + TGUG × 0.1960 + exhaustion × −0.0880 + depression × 0.0464 + albumin × −0.5343 + eGFR × 0.0175 + hemoglobin × −0.5204). Standardized discriminant coefficients of non-PC and PC patients are shown in Figure 3. In the non-PC patients, male sex, TGUG and age were positively associated with cancer, whereas handgrip, eGFR, albumin, and hemoglobin had a negative association (Figure 3A). Frailty discriminant score (FDS) showed a clear separation between controls and non-PC patients (Figure 3B). TGUG, albumin, age, handgrip, and BMI were associated positively with cancer, whereas eGFR and hemoglobin had a negative association in the PC patients (Figure 3C). FDS showed a clear separation between controls and PC patients (Figure 3D). The hit-rates of non-PC and PC patients were 95.2% and 93.4%, respectively.

### Clinical implication of FDS

**Figure 4:**
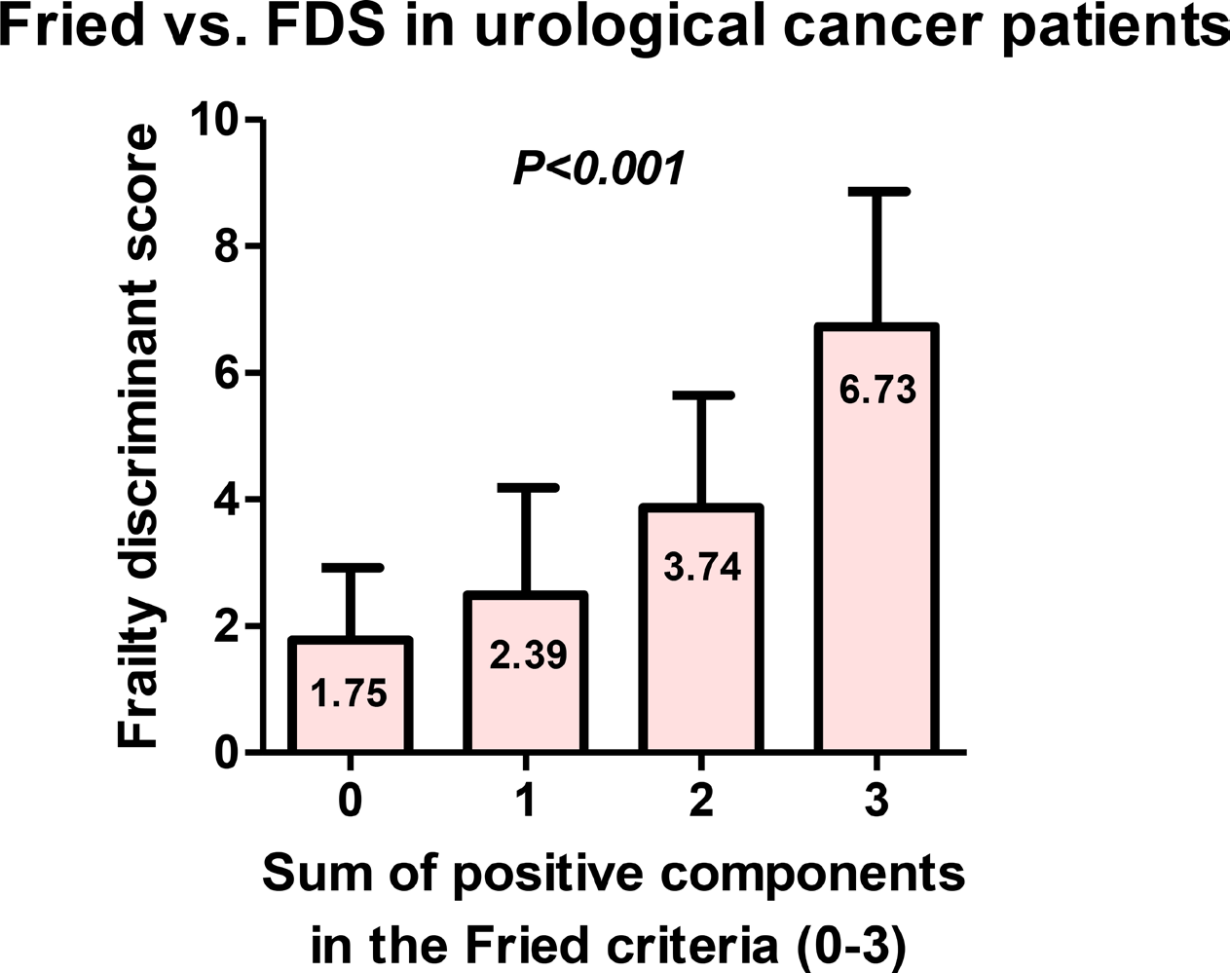
Association between the sum of three components in the Fried criteria and FDS Sum of positive components included handgrip strength (male < 30 kg, female < 18 kg), gait speed (TGUG >13 sec.), and exhaustion (CES-D) for conventional frailty evaluation (score 0–3). There were significant association between sum of positive components in the Fried criteria and FDS (*P* < 0.001, Kruskal–Wallis test).

Sum of positive components included handgrip strength (male < 30 kg, female < 18 kg), gait speed (TGUG > 13 sec.), and exhaustion (the Center for Epidemiologic Studies for Depression; CES-D) for conventional frailty evaluation (score 0–3). There were significant differences between the sum of three components in the Fried criteria and FDS (*P* < 0.001, Kruskal–Wallis test) (Figure 4).

**Figure 5:**
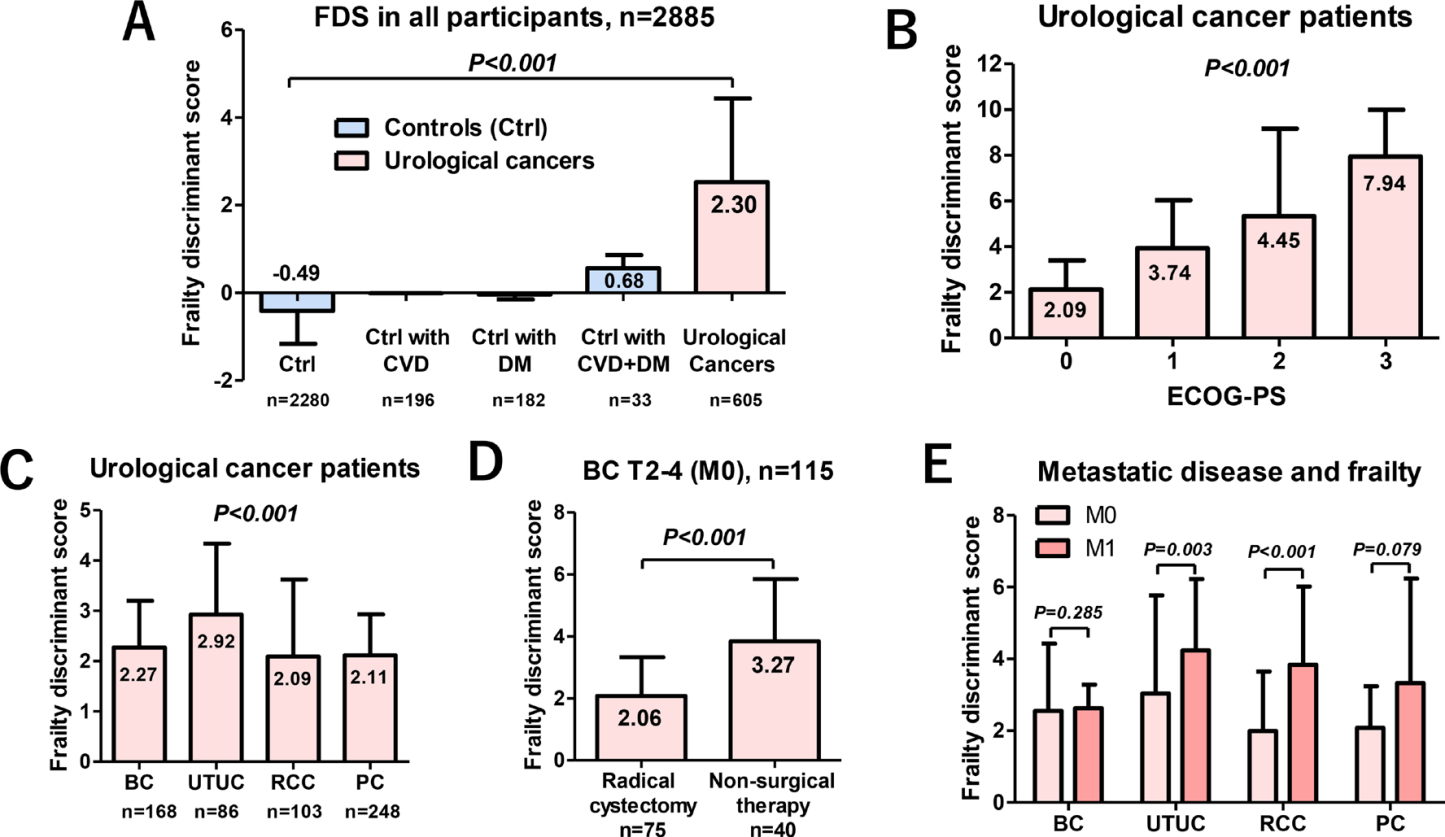
Characteristics of FDS for the controls and urological cancer patients The FDS in the urological cancer patients was significantly higher than that of the controls (*P* < 0.001). Among the controls, those with both CVD and DM had a significantly higher FDS than others (*P* < 0.001; **A**). FDS had a significant association with ECOG-PS in urological cancer patients (*P* < 0.001, Kruskal–Wallis test; **B**). Median FDSs were significantly different among the urological cancer patients (*P* < 0.001; **C**). Among patients with muscle-invasive BC, a significantly higher FDS was observed in those who underwent nonsurgical therapy (3.27) than in those who underwent radical cystectomy (2.06; *P* < 0.001) (**D**). Patients with metastatic diseases had a significantly higher FDS than those with localized diseases, except for BC and PC (**E**).

Median FDS in the controls was −0.49. The median score among the controls who had CVD, DM, and CVD plus DM were −0.01, −0.07, and 0.68, respectively (Figure 5A). Median FDS of the urological cancer patients was 2.30, which was significantly higher than that of the controls (*P* < 0.001). There were significant differences between the Eastern Cooperative Oncology Group performance status (ECOG-PS) and FDS (*P* < 0.001, Kruskal–Wallis test; Figure 5B). Median FDS was significantly different among the urological cancer patients (*P* < 0.001; Figure 5C). Among patients with muscle-invasive BC, a significantly higher FDS was observed in those who underwent nonsurgical therapy (3.27) than in those who underwent radical cystectomy (2.06, *P* < 0.001; Figure 5D). Patients with metastatic diseases had a significantly higher FDS than those with localized diseases except in the cases of BC and PC (Figure 5E).

### Impact of FDS on prognosis

**Figure 6:**
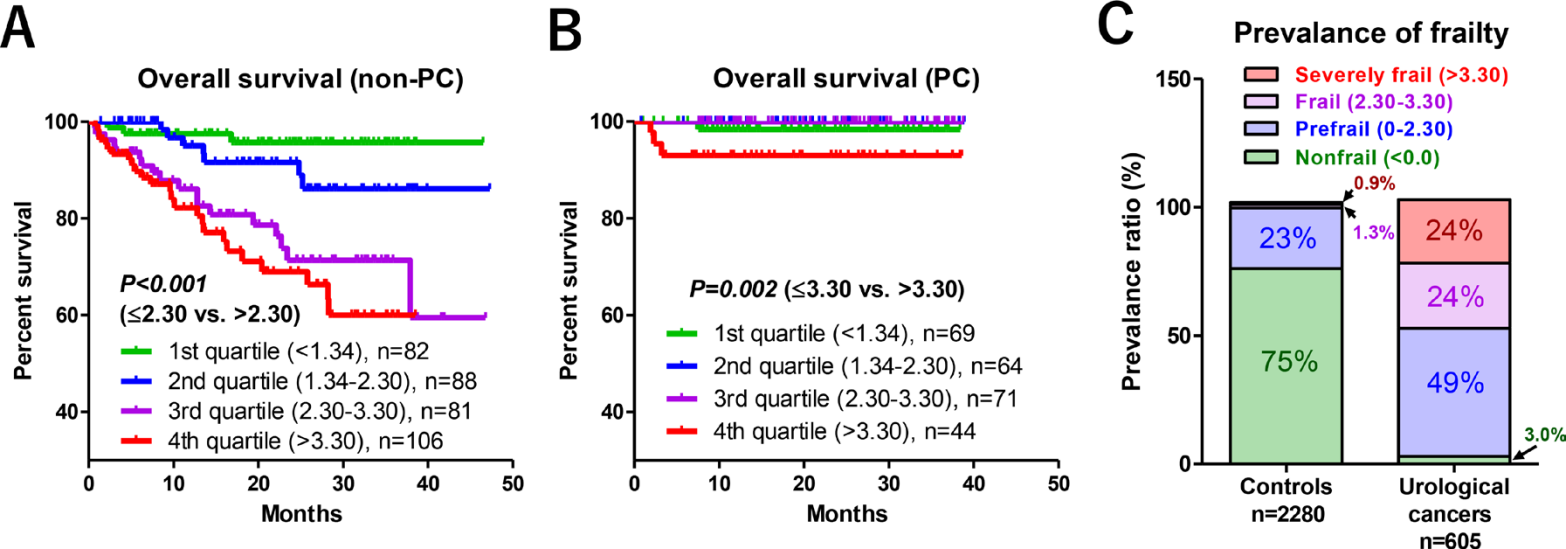
Relationship between FDS and overall survival, and prevalence of frailty When non-PC patients were stratified into four groups (first to fourth quartiles), overall survivals were significantly shorter in patients with third and fourth quartile FDSs (>2.30) compared to first and second quartile FDSs (≤2.30; *P* < 0.001; **A**). Frailty was associated with poor overall survival in PC patents when FDS was higher than 3.30 (**B**). When we defined frailty in the urological cancer patients as FDS > 2.30, the prevalence of frailty in urological cancer patients was 48% (**C**).

Among non-PC patients, overall survivals were significantly shorter in those with third and fourth quartile FDSs (>2.30) compared to those with first and second quartile FDSs (≤2.30; *P* < 0.001, Figure 6A). In PC patients, overall survivals were significantly shorter in patients with a higher score (>3.30) than in those with a lower score (Figure 6B). Multivariate Cox regression analysis revealed that metastatic disease in non-PC patients (*P* < 0.001; hazards ratio [HR], 9.34) and FDS > 2.30 remained independent factors for overall survival (*P* = 0.005; HR, 3.03; Table 3). When we defined frailty as FDS > 2.30, the prevalence of frailty in urological cancer patients was 48% (Figure 6C).

**Table 3:** Multivariate Cox regression analysis for overall survival in the non-PC patients (*n* = 357)

Variable	Factor	*P* value	HR	95.0% CI
Age	Continuous	0.367	1.02	0.98–1.05
Sex	Male	0.001	0.36	0.20–0.66
ECOG-PS	>1	0.034	2.13	1.06–3.39
Comorbidities (CVD or DM)	Positive	0.084	0.53	0.25–1.07
Metastatic disease	Positive	<0.001	9.34	5.12–17.1
Frailty discriminant score	>2.30	0.005	3.03	1.41–5.51

## DISCUSSION

This is the first study that compared frailty among urological cancer patients and evaluated the impact of quantitative frailty on prognosis. Our results suggested that urological cancer patients have impaired physical function, hypoalbuminemia, lower renal function, and anemia, and experience a higher rate of exhaustion and depression. On the other hand, renal function was significantly higher in patients with PC than in controls. Although we could not clearly address this reason, this is the first study to show the renal function might be not useful tools for frailty evaluation in PC patients. Further larger study is necessary to address the frailty in patients with PC. Based on these findings, we separately developed frailty discriminant formulas for non-PC and PC patients to distinguish between cancer patients and controls. Standardized discriminant coefficients showed that TGUG was associated positively with FDS, whereas higher eGFR and hemoglobin were associated negatively with FDS in patients with all types of urological cancer. The FDS clearly separates urological cancer patients from controls with higher hit-rates. In addition, FDS showed a positive association with frailty in controls with comorbidities (median FDS, 0.68). Higher FDS was associated significantly with poor ECOG-PS, although ECOG-PS included subjective bias of clinicians. It should be noted that the median FDSs in patients undergoing radical cystectomy and nonsurgical therapy (systemic chemotherapy and/or radiation) were 2.06 and 3.27, which suggests that FDS may support the clinical decision for radical cystectomy. These finding suggested that FDS may reflect clinical sensation for frailty. Of urological cancers, UTUC had the highest FDS (2.92), followed by BC (2.27), PC (2.11), and RCC (2.09). These results might be influenced by the highest age of UTUC patients (median, 73 years old) in our study. However, the influence of age on FDS in non-PC group was small (coefficient of age; 0.0771). Therefore, not only age but other frailty parameters strongly influenced on FDS in patients with UTUC. Patients with metastatic disease had a significantly higher FDS than those with localized disease, except for BC and PC, which might be due to fewer number of patients with metastases in the BC and PC groups (7.7% and 12%, respectively) than in UTUC and RCC groups (28% and 27%, respectively). However, further studies are needed to clarify the impact of metastatic disease on FDS.

The association of FDS with prognosis should be noted. In non-PC patients, a higher FDS (>2.30) was associated with a significantly poorer overall survival than a lower FDS (≤2.30) without including clinical stages. The 3-year survival probabilities in the first, second, third, and fourth quartiles were 96%, 86%, 73%, and 61%, respectively, which was not the case with PC patients. Severe frailty (FDS > 3.30) was significantly associated with poor overall survival in PC patients. These results suggested that the impact of frailty on prognosis was different according to diseases, and the clinical implication of frailty should be adapted disease management. As suggested by a recent systematic review [10], the optimal frailty tools may change depending on diseases, and suitable tools should be used to optimize disease management. Although our findings need further validation, FDS may reflect clinical sensation for frailty and support the clinical decision making.

We developed a quantitative frailty assessment tool using simple frailty assessment items and basic clinical data. Simplified tools for frailty assessment are required because of time constraints and the need for specialized testing. Recent studies suggested that the key components of the Fried criteria (shrinking, gait speed, and handgrip strength) hold a similar predictive value as the full frailty assessment [13, 14], and basic blood biochemical tests, such as serum albumin, renal function, and hemoglobin, were reported to be significantly associated with postoperative complication and/or frailty [6, 13, 15–17]. Although our study could not include shrinking or body weight loss due to absence of data, our results suggested that a quantitative approach can potentially predict poor prognosis without using clinical stages. In addition, FDS > 2.30 remained a significant predictor of overall survival (*P* = 0.005, HR, 3.03) in non-PC patients after controlling for strong factors, such as presence of metastatic disease. Therefore, our next multicenter study (UMIN000028533) must validate the present results.

Limitations of our study include the small sample size; single-institution; selection bias according to age, sex, type of cancers, and clinical stage; limited number of frail evaluations; and other unmeasurable confounding factors that could not be controlled. We could not compare our result to established values, such as those of the five components in Fried criteria or the comprehensive value of frailty assessment due to lack of data. Frailty assessment performed at academic medical center is one limitation, and our results may not be generalized to other patient populations. Urological cancer patients were tested at the hospital. However, healthy controls were tested at the medical checkup institute. The location of the TGUG may have an influence on the results. We could not address the relationship between frailty and postoperative complications because of mixed patients undergoing surgical and nonsurgical therapy. Despite these limitations, to our knowledge we are the first to investigate the clinical implication of a quantitative frailty assessment tool on prognosis in urological cancer patients. Although further studies are needed, our findings enhanced the importance of frailty assessment in clinical practice using a quantitative tool in urological cancer patients.

## CONCLUSIONS

Our study showed that FDS was associated significantly with frailty and prognosis in urological cancer patients. The impact of frailty on prognosis was different in PC patients. A quantitative tool for frailty assessment can help patients and physicians make more informed decisions.

## MATERIALS AND METHODS

### Ethics statement

This study was performed according to the ethical standards of the Declaration of Helsinki and approved by the ethics review board of the Hirosaki University School of Medicine (authorization number, 2014-297). All participants provided written informed consent. This study was registered as a clinical trial (UMIN000025057).

### Patient selection

Between August 2013 and June 2017, 2778 consecutive patients were admitted to our university hospital. Of those, a prospective frailty assessment was done on 605 urological cancer patients, including those with BC, UTUC, RCC and PC. We included 2280 subjects from community-dwelling populations from the Iwaki Health Promotion Project [18–21] as controls who underwent frailty assessment.

### Variable evaluations

The variables of age, sex, BMI, ECOG-PS, CVD, DM, types of urological cancers, treatment modality, and clinical stage were recorded for all subjects. Routine laboratory investigations were conducted, including blood count, serum albumin levels, and renal function tests. Renal function was assessed according to eGFR using a modified version of the abbreviated Modification of Diet in Renal Disease Study formula for Japanese patients: eGFR mL/min/1.73 m^2^ = 194 × sCr^−1.094^ × age^−0.287^ (× 0.739, if female) [22].

### Frailty assessment

The subjects underwent frailty assessment at hospitalization. We assessed frailty via six items, including handgrip strength, gait speed, serum albumin, renal function, hemoglobin, and self-reported exhaustion and depression. Gait speed was measured by TGUG [6]. Self-reported exhaustion and depression were assessed in cancer patients and community-dwelling populations by the fatigue scale of the CES-D and vitality questionnaire of Health-Related Quality of Life, respectively. Answers, such as “all of the time” or “most of the time,” for questionnaires were positive.

Of five components (weight loss, handgrip strength, gait speed, exhaustion, physical activity) in the Fried criteria, we used three components including handgrip strength (male < 30 kg, female < 18 kg), gait speed (TGUG > 13 sec.), and exhaustion (CES-D positive) because our data did not include weight loss and physical activity. We compared association between the sum of positive components in the Fried criteria and FDS by Kruskal–Wallis test.

Discriminant analysis was performed to develop a frailty discriminant formula between controls and urological cancer patients, including age, sex, BMI, handgrip strength, TGUG, serum albumin, renal function, hemoglobin, exhaustion, and depression. The formula was developed using a discriminant coefficient. These coefficients can be used to calculate the discriminant score for a given case. The score is calculated in the same manner as a predicted value from a linear regression, using the standardized coefficients and the standardized variables. Due to the sex bias, we separately developed a frailty discriminant formula for PC and non-PC (BC, UTUC, and RCC) patients. Based on the formula, we calculated FDS in all 2885 participants. We compared FDS between controls and urological cancer patients. Controls for discriminant analysis for non-PC and PC patients included 2280 and 874 male subjects without cancer, respectively. The degree of frailty was classified according to quartiles of FDS: nonfrail (<0.0), prefrail (0.0 and first quartile), frail (second and third quartile), and severely frail (fourth quartile and higher). Prevalence of frailty in the controls and urological cancer patients was evaluated based on these criteria.

### Statistical analysis

Statistical analyses of clinical data were performed using SPSS ver. 24.0 (IBM, Inc., Armonk, NY, USA) and GraphPad Prism 5.03 (GraphPad Software, San Diego, CA, USA). Categorical variables were compared using Fisher’s exact test or the χ^2^ test. Quantitative variables were expressed as means ± standard deviations. Differences between groups were compared using Student’s *t*-test for normally distributed data or the Mann–Whitney *U* test for nonnormally distributed data. Differences among three or more groups was analyzed using Kruskal–Wallis test. Differences were considered significant at *P* < 0.05. To select appropriate controls from the 2280 community-dwelling individuals, we compared TGUG, handgrip strength, serum albumin, eGFR, hemoglobin, and exhaustion and depression between controls and each urological cancer patient using propensity score matching as described previously [21, 23]. Propensity scores were calculated using logistic analysis (SPSS ver. 24), and they accounted for age, sex, BMI, and presence of comorbidities (DM and/or CVD). Two controls and one urological cancer patient with a score within 0.03 points of each other were selected as a paired group. The frailty discriminant formula between the controls and urological cancer patients was calculated by discriminant analysis. Overall survival was evaluated by the Kaplan-Meier method and log-rank test. Multivariate Cox regression analysis for independent predictor for overall survival in non-PC patients was performed including age, sex, ECOG-PS, comorbidities (CVD or DM), metastatic disease, and FDS.

## Abbreviations

BC: bladder cancer
UTUC: upper tract urothelial carcinoma
RCC: renal cell carcinoma
PC: prostate cancer
CVD: cardiovascular disease
DM: diabetes mellitus
TGUG: the timed get-up and go test
FDS: Frailty discriminant score
CES-D: the Center for Epidemiologic Studies for Depression
ECOG-PS: Eastern Cooperative Oncology Group Performance Status
eGFR: estimated glomerular filtration rate
HR: hazard ratio
